# Decision-to-delivery interval and neonatal outcomes in intrapartum umbilical cord prolapse

**DOI:** 10.1186/s12884-023-05788-y

**Published:** 2023-06-22

**Authors:** Ohad Houri, Asnat Walfisch, Adi Shilony, Hadas Zafrir-Danieli, Natav Hendin, Ran Matot, Inbal Navon, Eran Hadar

**Affiliations:** 1 Helen Schneider Hospital for Women, Rabin Medical Center – Beilinson Hospital, 39 Jabotinsky St., 4941492 Petach Tikva, Israel; 2 Sackler Faculty of Medicine, Tel Aviv University, Tel Aviv, Israel

**Keywords:** Cord arterial blood pH, Cord prolapse, Decision-to-delivery interval

## Abstract

**Background:**

Rapid delivery is important in cases of umbilical cord prolapse to prevent hypoxic injury to the fetus/neonate. However, the optimal decision-to-delivery interval remains controversial.

**Objective:**

The aim of the study was to investigate the association between the decision-to-delivery interval in women with umbilical cord prolapse, stratified by fetal heart rate pattern at diagnosis, and neonatal outcome.

**Study design:**

The database of a tertiary medical center was retrospectively searched for all cases of intrapartum cord prolapse between 2008 and 2021. The cohort was divided into three groups according to findings on the fetal heart tracing at diagnosis: 1) bradycardia; 2) decelerations without bradycardia; and 3) reassuring heart rate. The primary outcome measure was fetal acidosis. The correlation between cord blood indices and decision-to-delivery interval was analyzed using Spearman’s rank correlation coefficient.

**Results:**

Of the total 103,917 deliveries performed during the study period, 130 (0.13%) were complicated by intrapartum umbilical cord prolapse. Division by fetal heart tracing yielded 22 women (16.92%) in group 1, 41 (31.53%) in group 2, and 67 (51.53%) in group 3. The median decision-to-delivery interval was 11.0 min (IQR 9.0–15.0); the interval was more than 20 min in 4 cases. The median cord arterial blood pH was 7.28 (IQR 7.24–7.32); pH was less than 7.2 in 4 neonates. There was no correlation of cord arterial pH with decision-to-delivery interval (Spearman’s *Ρ* =  − 0.113; *Ρ* = 0.368) or with fetal heart rate pattern (Spearman’s *Ρ* = .425; *Ρ* = .079, *Ρ* =  − .205; *Ρ* = .336, *Ρ* =  − .324; *Ρ* = .122 for groups 1–3, respectively).

**Conclusion:**

Intrapartum umbilical cord prolapse is a relatively rare obstetric emergency with an overall favorable neonatal outcome if managed in a timely manner, regardless of the immediately preceding fetal heart rate. In a clinical setting which includes a high obstetric volume and a rapid, protocol-based, response, there is apparently no significant correlation between decision-to-delivery interval and cord arterial cord pH.

**Supplementary Information:**

The online version contains supplementary material available at 10.1186/s12884-023-05788-y.

## Introduction

Umbilical cord prolapse is a rare and unpredictable obstetric emergency with an incidence of 0.16–0.18% of live births [1, 2]. It occurs when the umbilical cord slips down in front of the presenting part of the fetus, into the cervical canal, vagina, or beyond, resulting from the outward flow of amniotic fluid that carries the cord. Among the maternal and fetal factors that have been associated with the risk for cord prolapse are multiparity, malpresentation, polyhydramnios, and preterm delivery [3, 4].

Cord prolapse commonly follows rupture of the membranes and is often associated with obstetric procedures such as amniotomy during disengagement of the presenting part [5]. In some cases, it is identified by the care provider during vaginal examination on palpation of the pulsating cord to assess labor progress [5–7]. It might be diagnosed with an abrupt onset of bradycardia or heart rate decelerations in a fetus with a previously normal tracing. Cord compression by the fetal presenting part and umbilical cord arterial vasospasm may lead to fetal hypoxia and asphyxia. The degree of cord compression, the interval between cord prolapse and delivery, and the successful use of intrauterine resuscitation maneuvers all impact the risk of adverse neonatal outcomes [8]. The rate of reported perinatal mortality related to cord prolapse varies widely from 0 to 53% [9, 10].

Rapid delivery is therefore important in umbilical cord prolapse to prevent fetal death or hypoxic brain injury [11]. However, there is no consensus on the optimal decision-to-delivery interval (DDI). Previous studies reported a poor correlation between the DDI and umbilical cord arterial blood gas indices [12, 13] or adverse neonatal outcomes, and some reported paradoxical results [14]. These findings could be partly attributable to the small sample size of the studies [5, 7, 10] and partly to their basing the analysis on the DDI alone and not the actual duration of fetal hypoxia [15, 16]. Whether the fetal heart rate (FHR) tracing at the onset of cord prolapse plays a predictive role remains unclear.

The aim of the present study was to investigate whether the DDI in women with umbilical cord prolapse, stratified by type of FHR tracing at diagnosis, is correlated with fetal cord pH and adverse neonatal outcomes.

## Materials and methods

The database of a tertiary medical center was retrospectively reviewed for all deliveries that occurred between January 2008 and December 2021. Women with umbilical cord prolapse were identified by the International Classification of Diseases codes.

### Definitions

Clinically overt cord prolapse was defined as the descent of the umbilical cord in advance of the fetal presenting part throughout the cervical os, in the presence of ruptured membranes. Women with a cord (funic) presentation, defined as the cord preceding the fetal presenting part, either seen on ultrasound or palpated during digital examination, in the presence of intact membranes, were excluded.

### Treatment protocol

At our institution, laboring women are routinely monitored continuously by cardiotocography, which is interpreted according to American College of Obstetricians and Gynecologists (ACOG) guidelines [17]. Parturient women are vaginally examined periodically to assess labor progress. After the membranes rupture, whether spontaneously or artificially, and in all cases of an abnormal FHR tracing, a vaginal examination is performed by a midwife or an obstetrician. When umbilical cord prolapse is diagnosed, an immediate, per-protocol, sequence of events takes place. The physician/midwife manually elevates the presenting part, without attempting to restitute the prolapsed cord above the presenting part, and the urinary bladder is rapidly retro filled with saline. In parallel, rapid preparations for an emergency cesarean delivery are undertaken. Two experienced obstetricians, a pediatrician, a neonatologist, and an anesthesiologist are readily and continuously available on-site 24 h a day. Once the patient is relocated to the nearby obstetric operating room, the baby is delivered either via cesarean section or vacuum extraction in accordance with the obstetric circumstances. A sample of arterial blood from the umbilical cord is obtained in all cases immediately following delivery.

### Data collection

Data for the study were retrieved from the computerized medical records. All cases of umbilical cord prolapse in our delivery ward were reviewed according to ICD10 code 069.0. Demographic and labor/pregnancy-related parameters included maternal age, parity, fetal presentation, presence of polyhydramnios, rupture of membranes (spontaneous or artificial), cervical dilatation, head position at time of diagnosis, and FHR tracing. Neonatal parameters included birthweight, gestational age at delivery, admission to the neonatal intensive care unit (NICU), 1- and 5-min Apgar scores, umbilical cord arterial pH, asphyxia, and neonatal outcome. Severe neonatal outcome was defined as a composite of any of the following: asphyxia, seizures, respiratory distress syndrome (RDS), necrotizing enterocolitis (NEC), intraventricular hemorrhage (IVH), or neonatal death. The timing of all major events, including the onset of FHR abnormalities, diagnosis of cord prolapse, and delivery, was documented.

### Data analysis

Women were divided into three groups by type of FHR tracing at diagnosis of umbilical cord prolapse: group 1, fetal bradycardia (baseline FHR < 110 beats per minute for > 5 min); group 2, any FHR decelerations without bradycardia; group 3, normal FHR tracing. In all groups, the DDI was defined as the time from diagnosis of cord prolapse to delivery.

The primary outcome measure of the study was fetal acidosis (pH < 7.2) according to cord arterial blood gas indices.

### Ethical approval

The study was approved by the local institutional review board (approval no. 0132–22-RMC).

### Statistical analysis

Standard statistical analyses were performed using SAS software, version 34.0 (SAS Institute Inc., Cary, NC, USA). Categorical variables were summarized by number and percentage or median and interquartile range, and continuous variables, by mean and standard deviation. Values were compared between groups using chi-square test or analysis of variance (ANOVA), as appropriate; a probability value below 0.05 was considered significant. Correlation analyses between umbilical cord arterial blood gas indices and DDI were performed using Spearman test, and results were stratified according to FHR tracing group.

## Results

A total of 103,917 deliveries were performed during the study period of which 130 (0.13%) were complicated by intrapartum events of umbilical cord prolapse. Stratification according to the predefined FHR patterns yielded 22 women (16.92%) in group 1 (bradycardia), 41 (31.53%) in group 2 (decelerations), and 67 (51.53%) in group 3 (normal FHR tracing) (Table 1).

**Table 1:** Maternal demographics and delivery intervals in different groups of umbilical cord prolapse emergencies according to FHR at diagnosis

	Group 1^*^ (***n* = 22)**	**Group 2 (*n* = 41)**	**Group 3 (*n* = 67)**	***p*-value**
Age (years)	32.14 ± 4.21	30.51 ± 5.39	31.61 ± 5.02	0.396
Gestational age (weeks)	37 + 6 (± 2.3days)	37 + 5(± 2.7days)	37 + 4(± 1.3days)	0.868
Preterm delivery < 37 weeks	4 (18.2%)	5 (12.2%)	8 (11.0%)	0.738
Polyhydramnion	2 (9.1%)	3 (7.3%)	7 (10.4%)	0.862
Nulliparity	3 (13.6%)	8 (19.5%)	13 (19.4%)	0.815
Amniotomy	14 (63.6%)	21 (51.2%)	33 (49.3%)	0.412
Meconium-stained amniotic fluid	5 (22.7%)	4 (9.8%)	6 (9%)	0.196
Cervical dilation at diagnosis (cm)	4 [3.8–8]	3 [2.5–4.75]	4 [3-5]	0.344
General anesthesia	18 (81.8%)	32 (78%)	44 (65.67%)	0.132
Vaginal delivery	1 (4.5%)	5 (12.2%)	1 (4.5%)	0.256
Decision-to-delivery interval (minutes)	10 [7-13]	12 [9-17]	11 [9-15]	0.322
Decision-to-delivery interval > 20 min	1 (28m)	2 (31 m,21m)	1 (21m)	0.543

Data are presented as number (%), mean ± standard deviation or median [interquartile range]

^*^group 1, Bradycardia; group 2, Decelerations; group 3, Normal heart rate

The mean maternal age at delivery was 31 ± 5 years. Twenty-four women (19.08%) were nulliparous. The median gestational age at delivery was 37 + 6 weeks; 17 women (13%) gave birth before 37 gestational weeks. Cesarean delivery was performed in 123 patients (94.7%), among women who had cesarian section, 94 (76.42%) with general anesthesia and regional anesthesia in 29 (23.57%).The mean birthweight was 3060 ± 685 g. There was no statistically significant difference among the FHR tracing groups in maternal age, gestational age at delivery, parity, rate of polyhydramnios, cervical dilation at diagnosis, mode of delivery, use of general anesthesia, preterm birth, and birthweight (Table 2).

**Table 2:** Pregnancy outcomes in different groups of umbilical cord prolapse emergencies according to FHR at diagnosis

	Group 1^*^ (***n* = 22)**	**Group 2 (*n* = 41)**	**Group 3 (*n* = 67)**	***p*-value**
Neonatal birthweight (grams)	3020 ± 809	2995 ± 560	3112 ± 716	0.662
Umbilical cord arterial pH < 7.2	2 (7.18, 7.16)	2 (7.18, 6.99)	1 (7.14)	0.142
Neonatal intensive care unit admission	4(18.2%)	11(26.8%)	12(18.2%)	0.532
Severe neonatal outcome^a^	0	0	0	-
Mechanical ventilation	2 (10%)	0	3 (4.8%)	0.22
Neonatal sepsis	1 (5%)	2 (5.5%)	4 (6.5%)	0.969
Transient tachypnea of the newborn	1 (5%)	3 (8.6%)	3 (4.8%)	0.72
5 Minute Apgar score < 7	0	4 (9.8%)	3 (4.5%)	0.21

Data are presented as number (%), mean ± standard deviation or median [interquartile range]

^*^group 1, Bradycardia; group 2, Decelerations; group 3, Normal heart rate

^a^Severe neonatal outcome: asphyxia, seizures, respiratory distress syndrome, necrotizing enterocolitis, intraventricular hemorrhage, death

Seven women (5.3%) had a vaginal delivery, all by assisted vacuum extraction. Within this group, one case involved the use of assisted vacuum extraction due to bradycardia, while the remaining cases were categorized under the NRFHR group. Among these seven cases, three women were nullipara. For each of the seven cases, the duration of decision-to-delivery interval (DDI) was less than 10 min. All newborns had favorable outcomes, and the arterial pH measured above 7.3.

The median DDI of the whole cohort was 11.0 min (IQR 9.0–15.0 min); the DDI was more than 20 min in only 4 cases (21, 21, 28, and 31 min). The median cord arterial pH was 7.28 (IQR 7.24–7.32); pH was below 7.2 in 4 neonates (7.14, 7.18, 7.18, 6.99). There was no significant difference in the median DDI among the three FHR tracing groups (Table 1).

**Fig. 1:**
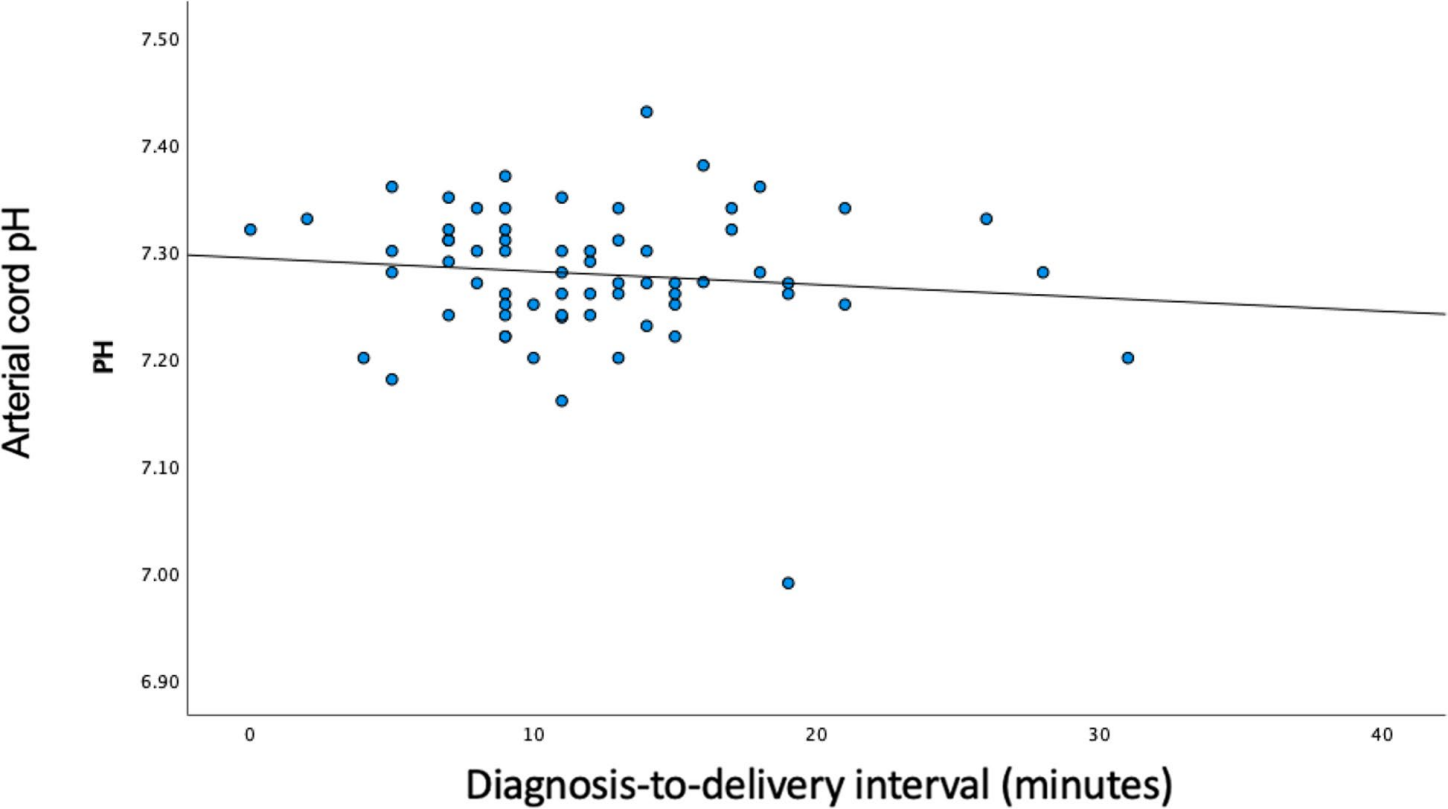
Correlation between cord arterial pH and diagnosis-to-delivery interval in intrapartum umbilical cord prolapse

**Fig. 2:**
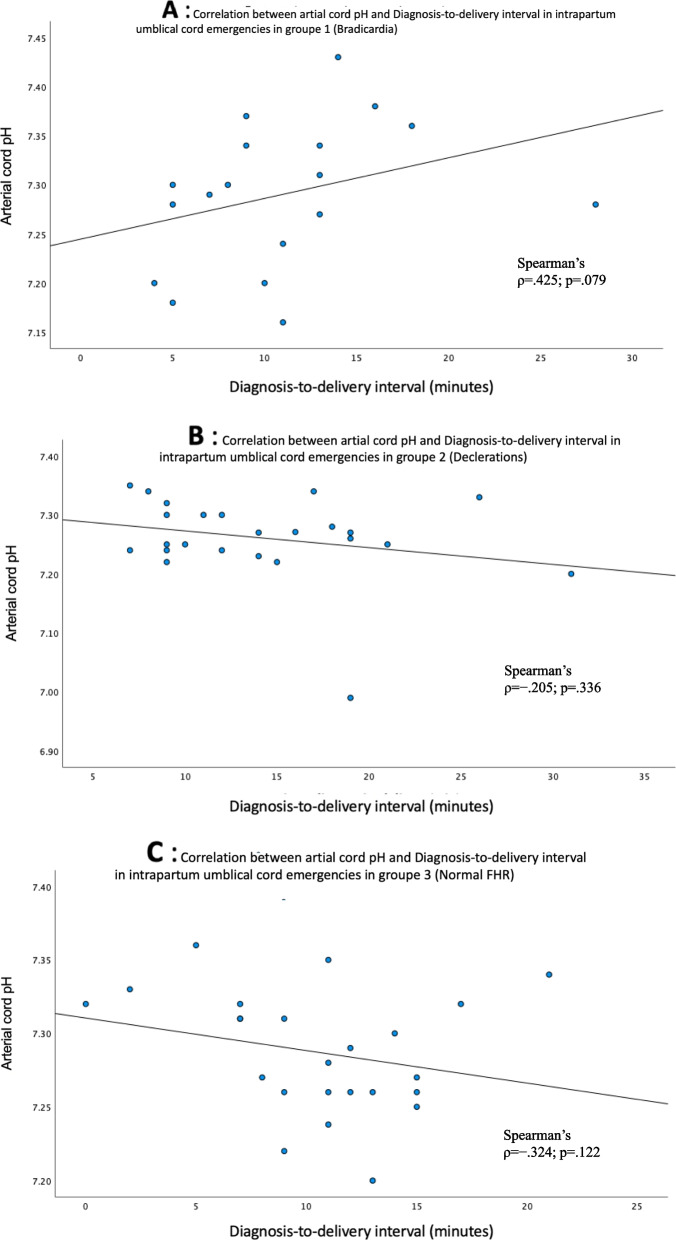
Correlations between cord arterial pH and diagnosis-to-delivery interval in intrapartum umbilical cord prolapse by precedent fetal heart rate tracing at diagnosis: **A** Group 1, fetal Bradycardia. **B** Group 2, intermittent fetal heart decelerations, (**C**) Groupe 3, normal fetal heart rate

On Spearman’s analysis, there was no correlation of cord arterial pH with the DDI (*Ρ* =  − 0.113, *Ρ* = 0.368) (Fig. 1) or with the FHR pattern (group 1: *Ρ* = 0.425, *P* = 0.079; group 2: *Ρ* =  − 0.205, *Ρ* = 0.336; group 3: *Ρ* =  − 0.324, *Ρ* = 0.122; Fig. 2). In group 1, an inverse association was observed between the cord arterial pH and the DDI, but it did not reach statistical significance.

Twenty-seven neonates (20.7%) were admitted to the NICU because of respiratory abnormalities (8 neonates), transient tachypnea of the newborn (8 neonates), respiratory distress syndrome (1 neonate), suspected neonatal sepsis in (7 neonates), and prematurity (3 neonates). The rate of NICU admission was similar in all three FHR groups (18.2%, 26.8%, 18.2% in groups 1, 2 and 3, respectively; *Ρ* = 0.532). There were no cases of severe neonatal outcome (fetal or neonatal death, intraventricular hemorrhage, asphyxia, necrotizing enterocolitis, seizures).

## Discussion

This retrospective cohort study found that among all deliveries complicated by umbilical cord prolapse in a tertiary medical center over a 12-year period, neonatal outcomes were generally favorable. There was no significant correlation between the DDI and umbilical cord arterial pH, regardless of findings on the FHR tracing immediately preceding the diagnosis of cord prolapse.

Although earlier studies reported a prevalence of 0.4–0.6% for umbilical cord prolapse, [1, 3] larger and more recent series showed lower rates of 0.16–0.18% [2, 4]. The decrease may be related to the increased use of ultrasound in the third trimester and delivery room, reduction in multiparity rates, and rising rates of cesarean delivery [18, 19]. In the present cohort, the prevalence was even lower (0.13%). We hypothesize that this finding may be explained by the same worldwide trends, and specifically, our routine intrapartum use of ultrasound which makes it possible to diagnose funic presentation prior to membrane rupture.

Previous studies recorded a wide range of perinatal mortality in cases of umbilical cord prolapse, from 0 to 53% [6, 9, 10, 20]. The largest study, consisting of 438 women from Uganda [10], demonstrated an alarming 53.5% perinatal mortality when the DDI was more than 60 min and 12.1% when the DDI was less than 30 min. Kaymak et al. [21], in a study of 98 cases of umbilical cord prolapse, found that a delivery interval of more than 10 min predicted adverse neonatal outcome. In the present cohort, there were no cases of perinatal mortality or severe morbidity among 130 cases of umbilical cord prolapse, and all neonates had good outcomes. Our favorable results are probably related to the hospital setting and the readily available trained staff and necessary equipment. Moreover, all parturient women in our center are continuously monitored, and the medical-obstetrical team is well-trained and experienced [22].

According to the umbilical cord prolapse guidelines of the Royal College of Obstetricians and Gynecologists (RCOG), a 30-min DDI is the acknowledged target for emergency caesarean delivery [23]. The reported average interval in UK maternity departments between decision and childbirth in cases of fetal concern was 30–40 min [24]. The National Sentinel Caesarean Section Audit [25] of cases with cord prolapse documented a median DDI of 17 min, with 75% of births performed within less than 26 min (IQR 12–26 min). In the present study, the median DDI was 11 min, considerably shorter than previously reported [24–26]. The difference may be again explained by the study setting of a high- tertiary medical center and the immediate availability of designated personnel and operating room at all times.

The 30-min rule for DDI when emergency cesarean delivery is indicated has become common practice, adopted by many professional associations [11, 27, 28]. However, there is still no consensus on the optimal DDI in these cases because of the poor correlation reported between the DDI and umbilical cord arterial blood pH and other neonatal outcomes [23, 29]. On the one hand, Chauhan et al. [30] found a greater frequency of adverse neonatal outcome when the DDI was more than 30 min. On the other hand, in cases of a shorter interval, Leung et al. [12] and Kamoshita et al. [13] failed to demonstrate any association between the DDI or low cord blood pH and adverse neonatal outcome. Faiz et al. [14] even reported an inverse result of improved Apgar scores at 5 min with DDIs longer than 20 min.

One of the major reasons for the large variability in previously reported results is the small size of many of the studies [5, 7, 8]. The lack of a consistent correlation between the DDI and neonatal outcomes may also be due to the fetal condition as reflected by the immediately preceding heart rate pattern [31]. Both the actual cord prolapse as well as the onset of fetal hypoxia may occur well before the diagnosis of cord prolapse is made. In order to overcome this potential bias, we analyzed fetal outcomes according to fetal heart rate tracing patterns at the time of cord prolapse diagnosis. Our finding that less than half the cases were accompanied by an abnormal FHR tracing (groups 1 and 2) can easily explain the lack of correlation between the DDI and neonatal status. If the fetus is not compromised during cord prolapse, the DDI loses importance. Similar to our findings, Koonings et al. [32] found that of 89 cases of cord prolapse in women being monitored electronically, only 66% had abnormalities of different severities in the FHR tracing.

However, we could not demonstrate a correlation between the DDI and cord arterial pH in any of the FHR tracing groups. In cases of fetal bradycardia in general, it is intuitively clear that the DDI is crucial. Accordingly, in a cohort of women with umbilical cord prolapse, Wong et al. [16] found a significant correlation between cord arterial pH and the bradycardia-to-delivery interval, but significance was not maintained when FHR monitoring demonstrated decelerations only. Similar results were shown by Leung et al. [12] Our finding that the DDI did not significantly correlate with neonatal acidosis even in the fetal bradycardia group when the outcome was inversed. This might probably a consequence of the very rapid response to the cord prolapse and the small size of the bradycardia group. When a case of cord prolapse is managed per protocol by an experienced and trained team, a difference of several minutes appears to have a major impact on neonatal outcome. If regional anesthesia can be achieved within minutes, it should be considered even in this emergency setting.

### Strengths and limitations

The major strength of the study is the relatively large sample and the inclusion of the entire population of women attending a single high-volume obstetric department in which all cases are managed with the identical delivery protocols, including intrapartum cord prolapse emeregncy. The study was limited by the retrospective design, which may preclude its general applicability of the findings, although it is not feasible to conduct a randomized or prospective study in such clinical scenarios. The fetal bradycardia group included only 22 women which made it difficult to draw conclusions. In addition, clinical follow-up was limited, and only early neonatal complications were recorded and analyzed.

## Conclusion

Intrapartum umbilical cord prolapse is a rare obstetric emergency. The FHR tracing may show bradycardia, recurrent decelerations, or commonly, a normal heart rate. If cord prolapse is managed in a timely manner, favorable neonatal outcomes are expected, regardless of the immediately preceding FHR tracing.

## Supplementary Information

**Additional file 1.**

## Data Availability

The datasets generated and analysed during the current study are not publicly available due ethical committee regulations, but are available from the corresponding author on reasonable request.

## References

[1] Murphy DJ, MacKenzie IZ (1995). The mortality and morbidity associated with umbilical cord prolapse. Br J Obstet Gynaecol.

[2] Gannard-Pechin E, Ramanah R, Cossa S, Mulin B, Maillet R, Riethmuller D (2012). Umbilical cord prolapse: a case study over 23 years. J Gynecol Obstet Biol Reprod.

[3] Uygur D, Kiş S, Tuncer R, Ozcan FS, Erkaya S (2002). Risk factors and infant outcomes associated with umbilical cord prolapse. Int J Gynaecol Obstet.

[4] Behbehani S, Patenaude V, Abenhaim HA (2016). Maternal risk factors and outcomes of umbilical cord prolapse: a population-based study. J Obstet Gynaecol Can.

[5] Gabbay-Benziv R, Maman M, Wiznitzer A, Linder N, Yogev Y (2014). Umbilical cord prolapse during delivery - risk factors and pregnancy outcome: a single center experience. J Matern Fetal Neonatal Med.

[6] Kahana B, Sheiner E, Levy A, Lazer S, Mazor M (2004). Umbilical cord prolapse and perinatal outcomes. Int J Gynaecol Obstet.

[7] Khan RS, Naru T, Nizami F (2007). Umbilical cord prolapse-a review of diagnosis to delivery interval on perinatal and maternal outcome. J Pak Med Assoc.

[8] Katz Z, Shoham Z, Lancet M, Blickstein I, Mogilner BM, Zalel Y (1988). Management of labor with umbilical cord prolapse: a 5-year study. Obstet Gynecol.

[9] Dilbaz B, Ozturkoglu E, Dilbaz S, Ozturk N, Sivaslioglu AA, Haberal A (2006). Risk factors and perinatal outcomes associated with umbilical cord prolapse. Arch Gynecol Obstet.

[10] Wasswa EW, Nakubulwa S, Mutyaba T (2014). Fetal demise and associated factors following umbilical cord prolapse in Mulago hospital, Uganda: a retrospective study. Reprod Health.

[11] Weiner E, Bar J, Fainstein N (2014). The effect of a program to shorten the decision-to-delivery interval for emergent cesarean section on maternal and neonatal outcome. Am J Obstet Gynecol.

[12] Leung TY, Chung PW, Rogers MS, Sahota DS, Lao TT, Hung Chung TK (2009). Urgent cesarean delivery for fetal bradycardia. Obstet Gynecol.

[13] Kamoshita E, Amano K, Kanai Y (2010). Effect of the interval between onset of sustained fetal bradycardia and cesarean delivery on long-term neonatal neurologic prognosis. Int J Gynaecol Obstet.

[14] Faiz SA, Habib FA, Sporrong BG, Khalil NA (2003). Results of delivery in umbilical cord prolapse. Saudi Med J.

[15] Kelly R, Ramaiah SM, Sheridan H (2018). Dose-dependent relationship between acidosis at birth and likelihood of death or cerebral palsy. Arch Dis Child Fetal Neonatal Ed.

[16] Wong L, Tse WT, Lai CY (2021). Bradycardia-to-delivery interval and fetal outcomes in umbilical cord prolapse. Acta Obstet Gynecol Scand.

[17] ACOG Practice Bulletin No (2009). 106: Intrapartum fetal heart rate monitoring: nomenclature, interpretation, and general management principles. Obstet Gynecol.

[18] Copson S, Calvert K, Raman P, Nathan E, Epee M (2017). The effect of a multidisciplinary obstetric emergency team training program, the in time course, on diagnosis to delivery interval following umbilical cord prolapse - A retrospective cohort study. Aust N Z J Obstet Gynaecol.

[19] Santana EFM, Castello RG, Passos MET, Ribeiro GCF, Araujo JE (2022). How to reach the best ultrasound performance in the delivery room. Rev Bras Ginecol Obstet.

[20] Lin MG (2006). Umbilical cord prolapse. Obstet Gynecol Surv.

[21] Kaymak O, Iskender C, Ibanoglu M, Cavkaytar S, Uygur D, Danisman N (2015). Retrospective evaluation of risk factors and perinatal outcome of umbilical cord prolapse during labor. Eur Rev Med Pharmacol Sci.

[22] Siassakos D, Hasafa Z, Sibanda T (2009). Retrospective cohort study of diagnosis-delivery interval with umbilical cord prolapse: the effect of team training. BJOG.

[23] Royal College of Obstetricians and Gynaecologists/Royal College of Anesthetists Practice Bulletin No. 11. Classification of urgency of cesarean section – a continuum of risk. Good Practice No. 11. London: RCOG Press; April 2010. https://www.oaa-anaes.ac.uk/assets/_managed/editor/File/RCoA/Good%20Practice%2011%20CS%20FINAL.pdf.

[24] National Collaborating Centre for Women's and Children's Health (UK). Intrapartum care: care of healthy women and their babies during childbirth. London: RCOG Press; Sep 2007. 21250397

[25] Thomas J, Paranjothy S (2001). Royal College of Obstetricians and Gynecologists Clinical Effectiveness Support Unit. The National Sentinel Caesarean Section Audit Report.

[26] Tan WC, Tan LK, Tan HK, Tan AS (2003). Audit of 'crash' emergency caesarean sections due to cord prolapse in terms of response time and perinatal outcome. Ann Acad Med Singap.

[27] American College of Obstetricians and Gynecologists Committee on Professional Standards: Standards for Obstetric-Gynecologic Services, 7th ed. Washington, DC: American College of Obstetricians and Gynecologists; 1989.

[28] Royal College of Obstetricians and Gynecologists (1995). Report of a joint working group: organization standards for maternity services.

[29] MacKenzie IZ, Cooke I (2002). What is a reasonable time from decision-to-delivery by caesarean section? Evidence from 415 deliveries. BJOG.

[30] Chauhan SP (1997). Cesarean section for suspected fetal distress. Does the decision- delivery time make a difference?. J Reprod Med.

[31] Bloom SL, Leveno KJ, Spong CY (2006). National institute of child health and human development maternal-fetal medicine units network. decision-to-incision times and maternal and infant outcomes. Obstet Gynecol.

[32] Koonings PP, Paul RH, Campbell K (1990). Umbilical cord prolapse, a contemporary look. J Reprod Med.

