# Supplementary figures and images for: Decision-to-delivery interval and neonatal outcomes in intrapartum umbilical cord prolapse
Source: BMC Pregnancy Childbirth. 2023 Jun 22;23:463. doi: 10.1186/s12884-023-05788-y (PMC10288770; doi:10.1186/s12884-023-05788-y)

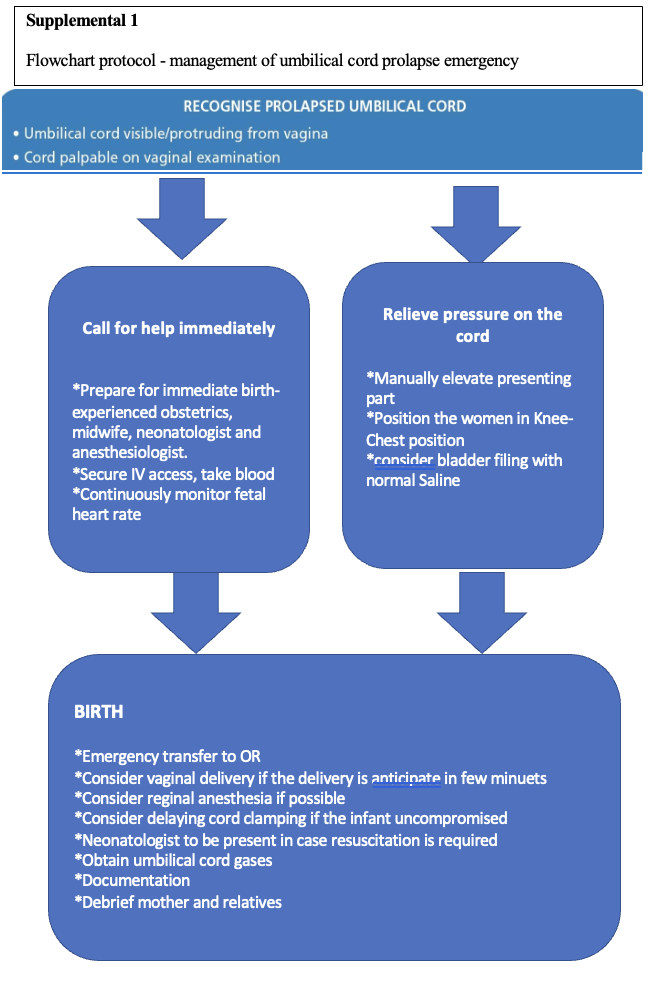

Supplement: Supplementary file 1 — Additional file 1. [file 12884_2023_5788_MOESM1_ESM.docx]
